# Association between dietary habits and incident thyroid cancer: A prospective cohort study

**DOI:** 10.3389/fnut.2023.1104925

**Published:** 2023-02-15

**Authors:** Linh Thi Dieu Nguyen, Madhawa Gunathilake, Jeonghee Lee, Jeongseon Kim

**Affiliations:** Department of Cancer Biomedical Science, National Cancer Center Graduate School of Cancer Science and Policy, Goyang-si, Republic of Korea

**Keywords:** thyroid cancer, dietary habits, dairy consumption, meal duration, prospective cohort study, Korean population

## Abstract

**Background:**

In addition to the thyroid cancer (TC) risk from lifestyle and environmental factors such as radiation exposure, some studies have indicated that diet may affect TC development; however, previous findings are inconsistent. The objective of our study was to investigate the association between dietary habits and TC risk in a Korean population.

**Materials and methods:**

A total of 13,973 participants were selected after excluding ineligible subjects from the Cancer Screenee Cohort at National Cancer Center in Korea from October 2007 to December 2021. Participants were followed until May 2022 to identify incident TC cases. Information on dietary habits and general characteristics was collected using a self-report questionnaire administered at enrollment without keeping track of changes in eating habits during the follow-up period. A Cox proportional hazards model was used to determine the hazard ratio (HR) and 95% confidence interval (CI) of TC risk for each dietary factor.

**Results:**

A total of 138 incident TC cases were identified during the median follow-up period of 7.6 years. Of the 12 dietary habits evaluated, only two habits showed significant associations with TC. A significantly decreased TC risk was found among participants who consumed milk and/or dairy products 5 or more days a week [adjusted HR (aHR), 0.58; 95% CI, 0.39–0.85]. Notably, a stronger protective effect of dairy consumption was observed in participants aged ≥ 50 years (aHR, 0.44; 95% CI, 0.26–0.75), in women (aHR, 0.53; 95% CI, 0.35–0.81), and in non-smokers (aHR, 0.60; 95% CI, 0.39–0.92). There was a reduced risk of TC in participants with meal durations longer than 10 min (aHR, 0.58; 95% CI, 0.41–0.83). However, this association was limited to individuals aged ≥ 50 years (aHR, 0.49; 95% CI, 0.31–0.79), women (aHR, 0.61; 95% CI, 0.41–0.90), and non-smokers (aHR, 0.62; 95% CI, 0.41–0.92).

**Conclusion:**

Our findings suggest that consuming milk and/or dairy products 5 or more days a week and having a meal duration longer than 10 min could be protective factors against TC, especially in individuals aged ≥ 50 years, women and non-smokers. Further prospective studies are needed to investigate the association of dietary intake with specific types of TC.

## Introduction

Recently, the incidence of thyroid cancer (TC) has increased worldwide; in 2020, TC was one of the ten most common types of cancer globally based on GLOBOCAN data (1). In South Korea, TC ranked as the most common cancer type in terms of the age-standardized incidence rate for both sexes in 2019 (42.9 per 100,000), although the rank of TC dropped due to controversy surrounding overdiagnosis in 2014 (2). Moreover, South Korea has the highest incidence rate of TC worldwide; in this country, TC cases are predominantly cases of papillary thyroid carcinoma (3, 4).

To date, the underlying causes of TC have remained unclear. Previous findings proposed that there were some potential factors, such as the environment and lifestyle, that may play a role in TC development (5, 6). Specifically, positive associations between TC risk and several other factors, including ionizing radiation, history of benign thyroid nodules/adenoma or goiter, and sex (female), have been reported (7).

Among the modifiable risk factors for TC, diet seems key to TC development (7, 8). Of them, there are both carcinogenic substances and antineoplastic agents, which are considered to impact TC risk (9). For instance, several studies have found that iodine deficiency or extreme iodine excess and high consumption of cruciferous vegetables containing goitrogens seem to be related to a high risk of TC (10–12). Conversely, dietary patterns rich in fresh fruits and vegetables were found to prevent TC risk (13). Nevertheless, the relationship between TC and dietary habits remains ambiguous. For example, while previous findings suggested that using multivitamin supplementation during a long-term period (> 10 years) decreased the risk of papillary TC (14), another study indicated that the risk of this type of TC is increased in women who consumed multivitamins for more than 10 years (15). Moreover, there is limited evidence of a relationship between dietary habits and TC risk specifically in Asian populations, including those in South Korea, due to inconclusive findings.

Previous studies have reported that papillary TC is the primary type of TC among Koreans (4), with the majority of patients younger than 50 years at diagnosis (16), and it peaked at the age of 50 years, especially among women (17), while women were predominant (64.8%) in our study. Additionally, Haymart indicated that 50 years is the age at which age-related worsening of TC prognosis occurs in women because of menopause, which alters the impact of estrogen or follicle-stimulating hormone/luteinizing hormone on TC development (18). Moreover, the median age of participants was close to 50 years in this study. Thus, it would be important to observe the association between dietary habits and the risk of TC stratified by age (< 50 or ≥ 50 years) and sex.

Additionally, several studies have implied that smoking status was a factor that reduced TC risk (19, 20). Haymart et al. suggested that higher serum thyroid-stimulating hormone (TSH) levels could be responsible for the development of TC (21). Smokers may have lower TSH concentrations than non-smokers (22). Another possible biological explanation may be related to the tobacco components. The stimulated nicotine receptors on immune cells may be involved in the decreased risk of Hashimoto’s thyroiditis (23). Another tobacco alkaloid, anatabine, also helped to reduce not only the incidence but also the severity of thyroiditis related to thyroglobulin (24), while a positive relationship between TC and chronic thyroiditis, such as Hashimoto’s thyroiditis, was found in some studies (25, 26). However, the relationship between eating habits and TC development stratified by smoking status is still unclear and needs to be evaluated.

To address this research gap, we carried out a prospective cohort study in South Korea, which has a high incidence of TC. We aimed to identify the association between some dietary habits and TC development and evaluate this relationship after stratification by age, sex, and smoking status (ever smokers, non-smokers) in a Korean population.

## Materials and methods

### Study population

**FIGURE 1:**
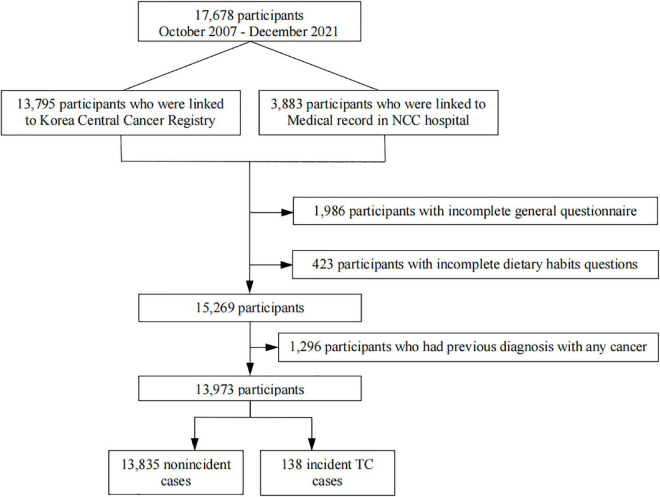
Flow chart of the study participants.

Our study analyzed data from participants recruited from the National Cancer Center (NCC) Cancer Screenee Cohort in South Korea from October 2007 to December 2021. Details regarding this cohort have been previously described (27). In short, a total of 17,678 participants were recruited, and they provided demographic information and underwent a health examination at the Center for Cancer Prevention and Detection of the NCC. Participants were followed until May 2022 to determine incident TC cases. The main exclusion criteria were as follows: incompleteness of the general questionnaire or dietary habit questions and previous diagnosis of any cancer. After the exclusion criteria were applied, 13,973 eligible participants were selected for inclusion in the final analysis (Figure 1). We acquired written informed consent from all participants and approval for the study protocol from the Institution Review Board of the NCC, South Korea (No. NCCNCS-07-077).

Incident cases of TC were identified by linkage to the 2019 Korea National Cancer Incidence Database of the Korea Central Cancer Registry and Medical Record from NCC Hospital in 2022. Biopsy was the final method used to diagnose most patients with TC. The identification of TC was based on the International Classification of Disease for Oncology (ICD-O), 10th revision (C73). An incident TC case was defined as a participant who developed primary TC after recruitment.

### Data collection

All participants enrolled in the Cancer Screenee Cohort of the NCC, South Korea, were inquired to fulfill a self-report questionnaire that collected demographic information, including age, sex, body mass index (BMI; in kg/m^2^; categorized as < 23, 23–< 25, or ≥ 25), smoking status (current smoker, ex-smoker, never smoker), alcohol consumption (current drinker, ex-drinker, never drinker), regular exercise (yes, no), first-degree family history of TC (FHTC; yes, no), monthly household income (in 10,000 won/month; categorized as < 200, 200–400, or ≥ 400), educational level (middle school, high school, college or more), and occupation (group 1: professionals and administrative management; group 2: office, sales, and service positions; group 3: agriculture and laborers; group 4: unemployed or other).

Information on the study participants’ dietary habits was assembled by using a cohort questionnaire from the Center for Cancer Prevention and Detection of the NCC, Korea. Participants were asked about dietary habits over the past year, which were evaluated using the following 12 items: (1) whether they consumed 3 meals/day for ≥ 5 days/week (yes, no), (2) whether their meal duration was ≥ 10 min (yes, no), (3) whether they ate meat and eggs ≥ 5 times/weeks (further classified as consuming no meat or consuming an amount of meat ≥ the size of 2 ping-pong balls and ≥ 1 egg, an amount of meat < the size of 2 ping-pong balls and < 1 egg), (4) whether they ate seafood ≥ 3 times/week (yes, no), (5) whether they ate tofu or soy milk ≥ 3 times/week (yes, no), (6) whether they ate vegetables, seaweed, mushrooms (except kimchi) with every meal (yes, no), (7) whether they ate fruits ≥ 5 days/week (yes, no), (8) whether they ate milk or dairy products ≥ 5 days/week (yes, no), (9) whether they ate ≥ 3 side dishes (except soup or kimchi) at every meal (yes, no), (10) whether they tasted salty when eating out (yes, no), (11) tend to eat salty food [yes, neutral (medium), no], and (12) frequency of grilled meat consumption [never, sometimes, often, N/A (not applicable)].

### Statistical analyses

For the data analysis, the χ^2^ test was used for categorical variables, and Student’s *t* test was used for continuous variables to compare the demographic characteristics between the incident TC cases and non-incident cases.

The participants’ person-years were calculated from the date they completed the self-report questionnaire to the date that they were diagnosed with cancer, they died or follow-up terminated (December 31, 2019 for subjects linked to Korea Central Cancer Registry; May 31, 2022 for those linked to medical record in the NCC Hospital), whichever happened first. We used a Cox proportional hazards regression model to assess the hazard ratio (HR) and 95% confidence interval (CI) of TC for each dietary habit, containing the 12 abovementioned dietary questions in all study populations. Additionally, we adjusted for significant variables based on the descriptive statistics of demographic information, including age, sex, smoking status, monthly household income, and occupation. Exceptionally, BMI was adjusted since it is considered a potential confounder based on previous findings (28, 29). Model 1 was adjusted for age and sex, and Model 2 was adjusted for age, sex, BMI, smoking status, household income and occupation. Moreover, we additionally adjusted for estrogen-related variables in women, particularly menarche age and menopausal status, in Model 2 when stratified by sex. All of our statistical analyses were executed in SAS software (version 9.4; SAS Institute, Cary, NC, USA), and the significance level was set at a two-sided *p* value less than 0.05.

### Sensitivity analysis

Since there was considerable variation in the participants with incident TC and non-incident cases, we carried out propensity score matching by age and sex variables to perform a sensitivity analysis in our study. Propensity score matching was performed based on the nearest neighbor method using the “MatchIt” package in R software version 4.2.0 (30). We conducted 1:2 ratio matching for TC case and non-cancer groups. Based on this matching, we were able to find 138 cancer cases matched with 276 non-cancer participants.

## Results

### General characteristics of the study participants

There were 138 incident TC cases (120 women and 18 men) identified during the median follow-up period of 7.6 years, and the sum of person-years observed was 103,613.23. The general characteristics of the study population are presented in Table 1. In the total population, subjects with incident TC were significantly younger than non-incident cases (50.9 ± 7.7 years vs. 53.3 ± 8.6 years; *p* = 0.001). Moreover, the proportion of incident TC cases in women was much higher than that in men (87.0 vs. 13.0%; *p* < 0.001). The subjects with TC had a lower proportion than those without TC in terms of current smoking status in all subjects (7.3 vs. 12.1%; *p* < 0.001), and a similar pattern was observed in those aged ≥ 50 years (5.3 vs. 11.0%; *p* = 0.003, respectively). In addition, compared to participants without TC, the individuals diagnosed with TC had lower employment rates not only among the entire population (*p* < 0.001) but also in the two age subgroups: those aged < 50 years (*p* = 0.006) and those aged ≥ 50 years (*p* = 0.029). Among participants older than 50 years, the proportion of incident TC cases with a low monthly income was lower than that among non-TC cases (*p* = 0.030).

**TABLE 1:** Baseline characteristics of the study participants.

Characteristic	Total			Age group					
				< 50 years			≥ 50 years		
	Non-incident cases (*n* = 13,835)	Incident TC cases (*n* = 138)	*p*-value^a^	Non-incident cases (*n* = 4,718)	Incident TC cases (*n* = 62)	*p*-value^a^	Non-incident cases (*n* = 9,117)	Incident TC cases (*n* = 76)	*p*-value^a^
Age, mean ± SD, years	53.3 ± 8.6	50.9 ± 7.7	**0.001**	44.0 ± 4.0	44.1 ± 4.1	0.867	58.1 ± 6.0	56.4 ± 4.9	**0.004**
**Sex (n, %)**									
Men	4904 (35.5)	18 (13.0)	**< 0.001**	1349 (28.6)	6 (9.7)	**0.001**	3555 (39.0)	12 (15.8)	**< 0.001**
Women	8931 (64.6)	120 (87.0)		3369 (71.4)	56 (90.3)		5562 (61.0)	64 (84.2)	
**BMI (kg/m^2^)**									
< 23	5847 (42.3)	60 (43.5)	0.822	2392 (50.7)	31 (50.0)	0.755	3455 (37.9)	29 (38.2)	0.942
23–< 25	3516 (25.4)	34 (24.6)		1008 (21.4)	15 (24.2)		2508 (27.5)	19 (25.0)	
≥ 25	4218 (30.5)	38 (27.5)		1245 (26.4)	14 (22.6)		2973 (32.6)	24 (31.6)	
Missing	254 (1.8)	6 (4.4)		73 (1.6)	2 (3.2)		181 (2.0)	4 (5.3)	
**Smoking status**									
Current smoker	1680 (12.1)	10 (7.3)	**< 0.001**	679 (14.4)	6 (9.7)	0.108	1001 (11.0)	4 (5.3)	**0.003**
Ex-smoker	2869 (20.7)	13 (9.4)		750 (15.9)	5 (8.1)		2119 (23.2)	8 (10.5)	
Never smoker	9266 (67.0)	114 (82.6)		3281 (69.5)	50 (80.7)		5985 (65.7)	64 (84.2)	
Missing	20 (0.1)	1 (0.7)		8 (0.2)	1 (1.6)		12 (0.1)	0 (0.0)	
**Alcohol consumption**									
Current drinker	7406 (53.5)	67 (48.6)	0.085	3013 (63.9)	37 (59.7)	0.142	4393 (48.2)	30 (39.5)	0.184
Ex-drinker	933 (6.7)	5 (3.6)		293 (6.2)	1 (1.6)		640 (7.0)	4 (5.3)	
Never drinker	5481 (39.6)	66 (47.8)		1404 (29.8)	24 (38.7)		4077 (44.7)	42 (55.3)	
Missing	15 (0.1)	0 (0.0)		8 (0.2)	0 (0.0)		7 (0.1)	0 (0.0)	
**Regular exercise**									
Yes	6763 (48.9)	54 (39.1)	0.163	1939 (41.1)	20 (32.3)	0.379	4824 (52.9)	34 (44.7)	0.439
No	5680 (41.1)	59 (42.8)		2175 (46.1)	29 (46.8)		3505 (38.4)	30 (39.5)	
Missing	1392 (10.1)	25 (18.1)		604 (12.8)	13 (21.0)		788 (8.6)	12 (15.8)	
**First-degree family history of TC**									
Yes	313 (2.3)	6 (4.4)	0.104	119 (2.5)	2 (3.2)	0.714	194 (2.1)	4 (5.3)	0.065
No	13384 (96.7)	131 (94.9)		4574 (97.0)	59 (95.2)		8810 (96.6)	74 (94.7)	
Missing	138 (1.0)	1 (0.7)		25 (0.5)	1 (1.6)		113 (1.2)	0 (0.0)	
**Marital status**									
Married	11338 (82.0)	113 (81.9)	0.710	3917 (83.0)	52 (83.9)	0.856	7421 (81.4)	61 (80.3)	0.770
Other	2197 (15.9)	20 (14.5)		724 (15.4)	9 (14.5)		1473 (16.2)	11 (14.5)	
Missing	300 (2.2)	5 (3.6)		77 (1.6)	1 (1.6)		223 (2.5)	4 (5.3)	
**Household income, in 10,000 Korean won/month**									
< 200	2671 (19.3)	17 (12.3)	0.100	549 (11.6)	9 (14.5)	0.143	2122 (23.3)	8 (10.5)	**0.030**
200–400	4591 (33.2)	45 (32.6)		1605 (34.0)	14 (22.6)		2986 (32.8)	31 (40.8)	
≥ 400	4508 (32.6)	52 (37.7)		2089 (44.3)	33 (53.2)		2419 (26.5)	19 (25.0)	
Missing	2065 (14.9)	24 (17.4)		475 (10.1)	6 (9.7)		1590 (17.4)	18 (23.7)	
**Educational level**									
Middle school	2005 (14.5)	15 (10.9)	0.365	173 (3.7)	2 (3.2)	0.572	1832 (20.1)	13 (17.1)	0.688
High school	4716 (34.1)	52 (37.7)		1547 (32.8)	24 (38.7)		3169 (34.8)	28 (36.8)	
College or more	6188 (44.7)	57 (41.3)		2816 (59.7)	33 (53.2)		3372 (37.0)	24 (31.6)	
Missing	926 (6.7)	14 (10.1)		182 (3.9)	3 (4.8)		744 (8.2)	11 (14.5)	
**Occupation**									
Group 1: Professionals and administrative management	3219 (23.3)	18 (13.0)	**< 0.001**	1601 (33.9)	11 (17.7)	**0.006**	1618 (17.8)	7 (9.2)	**0.029**
Group 2: Office, sales, and service positions	2843 (20.6)	33 (23.9)		1051 (22.3)	16 (25.8)		1792 (19.7)	17 (22.4)	
Group 3: Agriculture and laborers	1291 (9.3)	4 (2.9)		322 (6.8)	1 (1.6)		969 (10.6)	3 (4.0)	
Group 4: Unemployed or other	6018 (43.5)	78 (56.5)		1648 (34.9)	32 (51.6)		4370 (47.9)	46 (60.5)	
Missing	464 (3.4)	5 (3.6)		96 (2.0)	2 (3.2)		368 (4.0)	3 (4.0)	

^a^χ^2^ tests and *t* tests were used for categorical variables and continuous variables, respectively.

The baseline characteristics of the matched participants are reported in Supplementary Table 1. Compared to subjects without TC, a higher proportion of FHTC (*p* = 0.034) and higher levels of household income (*p* = 0.008), education (*p* = 0.023), and occupation (*p* = 0.014) were observed among those with incident TC. Significantly similar trends were found among individuals aged ≥ 50 years, except for education level.

### Associations between dietary habits and incident TC

Table 2 shows the relationship between dietary habits and TC risk in the total study population. A significantly decreased TC risk was found among participants who consumed milk and/or dairy products five or more days a week in Model 2 (aHR, 0.58; 95% CI, 0.39–0.85). Notably, a stronger protective effect was observed in participants older than 50 years in Model 2 (aHR, 0.44; 95% CI, 0.26–0.75). Nevertheless, this association was non-significant among individuals younger than 50 years old (Table 3). Additionally, we observed a reduced risk of TC in participants who had meal durations greater than 10 min (aHR, 0.58; 95% CI, 0.41–0.83). However, this association was limited to individuals aged ≥ 50 years (aHR, 0.49; 95% CI, 0.31–0.79).

**TABLE 2:** Hazard ratios (HRs) and 95% confidence intervals (CIs) of incident thyroid cancer (TC) related to dietary habits in total population.

Dietary habit	Total population			
	TC cases	Person-years	HR (95% CI) for Model 1	HR (95% CI) for Model 2
**Meal frequency: 3 meals/day for ≥ 5 days/week**				
No	30	23,902	1.00	1.00
Yes	104	78,438	1.30 (0.86–1.96)	1.34 (0.88–2.03)
**Meal duration ≥ 10 min**				
No	46	24,336	1.00	1.00
Yes	88	78,024	**0.57 (0.40–0.82)**	**0.58 (0.41–0.83)**
**Meat and eggs ≥ 5 times/week**				
No meat or amount of meat ≥ the size of 2 ping-pong balls and ≥ 1 egg	113	86,435	1.00	1.00
Amount of meat < the size of 2 ping-pong balls and < 1 egg	16	12,783	0.94 (0.56–1.58)	0.96 (0.57–1.62)
**Seafood ≥ 3 times/week**				
No	84	67,907	1.00	1.00
Yes	50	34,356	1.33 (0.94–1.89)	1.30 (0.91–1.85)
**Tofu or soy milk ≥ 3 times/week**				
No	77	57,292	1.00	1.00
Yes	57	44,989	0.95 (0.67–1.34)	0.94 (0.66–1.32)
**Vegetables, seaweed, mushrooms (except kimchi) every meal**				
No	60	50,896	1.00	1.00
Yes	74	51,407	1.21 (0.86–1.70)	1.21 (0.86–1.71)
**Fruits ≥ 5 days/week**				
No	65	46,706	1.00	1.00
Yes	67	55,600	0.79 (0.56–1.11)	0.75 (0.53–1.07)
**Milk or dairy products ≥ 5 days/week**				
No	97	63,878	1.00	1.00
Yes	35	38,275	**0.58 (0.39–0.86)**	**0.58 (0.39–0.85)**
**≥ 3 side dishes (except soup or kimchi) at every meal**				
No	26	22,586	1.00	1.00
Yes	106	79,763	1.35 (0.88–2.08)	1.33 (0.87–2.06)
**Taste salty when eating out**				
No	54	41,582	1.00	1.00
Yes	76	60,249	0.85 (0.60–1.21)	0.85 (0.60–1.22)
**Tend to eat salty food**				
Yes	12	10,357	1.00	1.00
Neutral (Medium)	96	70,530	1.03 (0.56–1.88)	1.02 (0.56–1.86)
No	24	21,434	0.83 (0.42–1.67)	0.84 (0.42–1.69)
**Grilled meat frequency**				
Never	90	58,004	1.00	1.00
Sometimes	38	36,621	0.70 (0.48–1.03)	0.71 (0.48–1.04)
Often	3	5,918	0.39 (0.12–1.22)	0.39 (0.12–1.23)
N/A (Not applicable)	1	1,615	0.41 (0.06–2.96)	0.44 (0.06–3.14)

Model 1 was adjusted for age and sex.

Model 2 was adjusted for age, sex, BMI, smoking status, household income, and occupation.

**TABLE 3:** Hazard ratios (HRs) and 95% confidence intervals (CIs) of incident thyroid cancer (TC) related to dietary habits by stratification of age.

Dietary habit	Age group							
	< 50 years				≥ 50 years			
	TC cases	Person-years	HR (95% CI) for Model 1	HR (95% CI) for Model 2	TC cases	Person-years	HR (95% CI) for Model 1	HR (95% CI) for Model 2
**Meal frequency: 3 meals/day for ≥ 5 days/week**								
No	17	11,229	1.00	1.00	13	12,673	1.00	1.00
Yes	43	25,477	1.21 (0.69–2.12)	1.23 (0.69–2.17)	61	52,961	1.33 (0.73–2.43)	1.39 (0.76–2.56)
**Meal duration ≥ 10 min**								
No	17	8,386	1.00	1.00	29	15,949	1.00	1.00
Yes	43	28,334	0.70 (0.40–1.23)	0.71 (0.40–1.26)	45	49,691	**0.49 (0.31–0.78)**	**0.49 (0.31–0.79)**
**Meat and eggs ≥ 5 times/week**								
No meat or amount of meat ≥ the size of 2 ping-pong balls and ≥ 1 egg	51	31,120	1.00	1.00	62	55,315	1.00	1.00
Amount of meat < the size of 2 ping-pong balls and < 1 egg	7	4,623	0.90 (0.41–1.97)	0.93 (0.42–2.05)	9	8,160	0.97 (0.48–1.95)	0.98 (0.49–1.98)
**Seafood ≥ 3 times/week**								
No	38	25,307	1.00	1.00	46	42,600	1.00	1.00
Yes	22	11,388	1.43 (0.84–2.41)	1.39 (0.82–2.36)	28	22,968	1.24 (0.77–1.98)	1.20 (0.75–1.93)
**Tofu or soy milk ≥ 3 times/week**								
No	40	21,948	1.00	1.00	37	35,343	1.00	1.00
Yes	20	14,744	0.73 (0.43–1.25)	0.73 (0.43–1.26)	37	30,245	1.14 (0.72–1.79)	1.11 (0.71–1.76)
**Vegetables, seaweed, mushrooms (except kimchi) every meal**								
No	28	18,430	1.00	1.00	32	32,466	1.00	1.00
Yes	32	18,285	1.12 (0.67–1.86)	1.14 (0.68–1.90)	42	33,122	1.28 (0.81–2.03)	1.27 (0.80–2.02)
**Fruits ≥ 5 days/week**								
No	31	18,698	1.00	1.00	34	28,008	1.00	1.00
Yes	28	17,992	0.82 (0.49–1.36)	0.78 (0.46–1.31)	39	37,609	0.74 (0.47–1.18)	0.71 (0.44–1.13)
**Milk or dairy products ≥ 5 days/week**								
No	42	24,795	1.00	1.00	55	39,083	1.00	1.00
Yes	17	11,870	0.81 (0.46–1.42)	0.80 (0.46–1.42)	18	26,405	**0.44 (0.26**–**0.76)**	**0.44 (0.26**–**0.75)**
**≥ 3 side dishes (except soup or kimchi) at every meal**								
No	11	9,333	1.00	1.00	15	13,253	1.00	1.00
Yes	48	27,367	1.67 (0.87–3.23)	1.65 (0.85–3.19)	58	52,396	1.09 (0.62–1.93)	1.07 (0.61–1.90)
**Taste salty when eating out**								
No	24	13,987	1.00	1.00	30	27,595	1.00	1.00
Yes	35	22,645	0.80 (0.48–1.35)	0.81 (0.47–1.37)	41	37,604	0.90 (0.56–1.45)	0.87 (0.54–1.40)
**Tend to eat salty food**								
Yes	3	3,486	1.00	1.00	9	6,871	1.00	1.00
Neutral (Medium)	44	25,734	1.74 (0.54–5.61)	1.74 (0.54–5.62)	52	44,796	0.80 (0.39–1.62)	0.78 (0.39–1.60)
No	12	75,023	1.60 (0.45–5.66)	1.72 (0.48–6.14)	12	13,931	0.58 (0.24–1.38)	0.57 (0.24–1.36)
**Grilled meat frequency**								
Never	39	18,434	1.00	1.00	51	39,570	1.00	1.00
Sometimes	19	15,567	0.62 (0.36–1.08)	0.62 (0.36–1.08)	19	21,053	0.82 (0.48–1.40)	0.83 (0.48–1.41)
Often	1	2,320	0.25 (0.03–1.83)	0.25 (0.03–1.81)	2	3,599	0.54 (0.13–2.22)	0.54 (0.13–2.24)
N/A (Not applicable)	0	329	NA	NA	1	1,285	0.58 (0.08–4.20)	0.63 (0.09–4.56)

Model 1 was adjusted for sex.

Model 2 was adjusted for sex, BMI, smoking status, household income, and occupation.

Table 4 presents the association between dietary habits and TC development after stratification by sex. After adjustment of estrogen-related variables shown in Supplementary Table 2, TC risk still exhibited inverse relationships with meal durations greater than 10 min and consuming milk and/or dairy products five or more days per week in women, with aHRs of 0.61 (95% CI, 0.41–0.90) and 0.53 (95% CI, 0.35–0.81), respectively.

**TABLE 4:** Hazard ratios (HRs) and 95% confidence intervals (CIs) of incident thyroid cancer (TC) related to dietary habits by stratification of sex.

Dietary habit	Men				Women			
	TC cases	Person-years	HR (95% CI) for Model 1	HR (95% CI) for Model 2	TC cases	Person-years	HR (95% CI) for Model 1	HR (95% CI) for Model 2
**Meal frequency: 3 meals/day for ≥ 5 days/week**								
No	2	6,121	1.00	1.00	28	17,781	1.00	1.00
Yes	16	30,171	1.94 (0.43–8.63)	1.99 (0.44–8.97)	88	48,267	1.26 (0.82–1.93)	1.28 (0.83–1.97)
**Meal duration ≥ 10 min**								
No	8	9,274	1.00	1.00	38	15,062	1.00	1.00
Yes	10	27,020	0.44 (0.17–1.12)	0.41 (0.16–1.03)	78	51,004	**0.60 (0.41**–**0.89)**	**0.61 (0.41**–**0.90)**
**Meat and eggs ≥ 5 times/week**								
No meat or amount of meat ≥ the size of 2 ping-pong balls and ≥ 1 egg	15	30,642	1.00	1.00	98	55,792	1.00	1.00
Amount of meat < the size of 2 ping-pong balls and < 1 egg	1	4,352	0.47 (0.06–3.58)	0.45 (0.06–3.45)	15	8,431	1.00 (0.58–1.73)	1.02 (0.59–1.77)
**Seafood ≥ 3 times/week**								
No	10	22,450	1.00	1.00	74	45,457	1.00	1.00
Yes	8	13,763	1.33 (0.52–3.38)	1.29 (0.50–3.32)	42	20,594	1.33 (0.91–1.95)	1.31 (0.89–1.92)
**Tofu or soy milk ≥ 3 times/week**								
No	9	21,035	1.00	1.00	68	36,257	1.00	1.00
Yes	9	15,206	1.44 (0.57–3.63)	1.29 (0.51–3.29)	48	29,783	0.89 (0.61–1.29)	0.87 (0.60–1.26)
**Vegetables, seaweed, mushrooms (except kimchi) every meal**								
No	6	18,390	1.00	1.00	54	32,505	1.00	1.00
Yes	12	17,890	2.11 (0.79–5.64)	1.92 (0.71–5.17)	62	33,517	1.12 (0.77–1.61)	1.11 (0.77–1.60)
**Fruits ≥ 5 days/week**								
No	9	20,212	1.00	1.00	56	26,494	1.00	1.00
Yes	9	16,026	1.39 (0.55–3.55)	1.15 (0.44–2.99)	58	39,574	0.72 (0.50–1.04)	0.69 (0.47–1.00)
**Milk or dairy products ≥ 5 days/week**								
No	12	24,383	1.00	1.00	85	39,496	1.00	1.00
Yes	6	11,798	1.08 (0.40–2.88)	0.97 (0.36–2.60)	29	26,478	**0.53 (0.35–0.80)**	**0.53 (0.35–0.81)**
**≥ 3 side dishes (except soup or kimchi) at every meal**								
No	3	5,810	1.00	1.00	23	16,776	1.00	1.00
Yes	15	30,438	0.99 (0.29–3.43)	0.98 (0.28–3.42)	91	49,324	1.40 (0.89–2.21)	1.39 (0.88–2.21)
**Taste salty when eating out**								
No	9	17,153	1.00	1.00	45	24,429	1.00	1.00
Yes	9	18,946	0.90 (0.36–2.26)	0.85 (0.33–2.16)	67	41,303	0.84 (0.58–1.23)	0.83 (0.57–1.22)
**Tend to eat salty food**								
Yes	3	4,742	1.00	1.00	9	5,615	1.00	1.00
Neutral (Medium)	13	24,371	0.84 (0.24–2.94)	0.83 (0.24–2.93)	83	46,159	1.09 (0.55–2.18)	1.07 (0.54–2.14)
No	2	7,145	0.45 (0.07–2.67)	0.43 (0.07–2.62)	22	14,289	0.93 (0.43–2.02)	0.92 (0.42–2.00)
**Grilled meat frequency**								
Never	11	17,218	1.00	1.00	79	40,787	1.00	1.00
Sometimes	7	15,455	0.66 (0.25–1.72)	0.68 (0.26–1.79)	31	21,265	0.71 (0.47–1.08)	0.71 (0.46–1.08)
Often	0	3,042	NA	NA	3	2,876	0.52 (0.16–1.64)	0.53 (0.17–1.68)
N/A (Not applicable)	0	434	NA	NA	1	1,181	0.46 (0.06–3.32)	0.51 (0.07–3.65)

Model 1 was adjusted for age.

Model 2 was adjusted for age, BMI, smoking status, household income and occupation and additionally adjusted for menarche age, and menopausal status in women.

Furthermore, we evaluated the impact of dietary habits on the development of TC stratified by smoking status. Both meal durations longer than 10 min and consumption of milk and dairy products more than 5 days per week decreased the risk of TC among those who never smoked, with aHRs of 0.62 (95% CI, 0.41–0.92) and 0.60 (95% CI, 0.39–0.92), respectively (Table 5).

**TABLE 5:** Hazard ratios (HRs) and 95% confidence intervals (CIs) of incident thyroid cancer (TC) related to dietary habits by stratification of smoking status.

Dietary habit	Smoking status							
	Ever smoker				Never smoker			
	TC cases	Person-years	HR (95% CI) for Model 1	HR (95% CI) for Model 2	TC cases	Person-years	HR (95% CI) for Model 1	HR (95% CI) for Model 2
**Meal frequency: 3 meals/day for ≥ 5 days/week**								
No	7	7,577	1.00	1.00	23	16,301	1.00	1.00
Yes	16	25,967	1.08 (0.42–2.79)	1.12 (0.43–2.89)	87	52,334	1.35 (0.85–2.15)	1.38 (0.87–2.20)
**Meal duration ≥ 10 min**								
No	10	8,609	1.00	1.00	36	15,690	1.00	1.00
Yes	13	24,916	0.45 (0.20–1.03)	0.44 (0.19–1.01)	74	52,983	**0.60 (0.40**–**0.89)**	**0.62 (0.41**–**0.92)**
**Meat and eggs ≥ 5 times/week**								
No meat or amount of meat ≥ the size of 2 ping-pong balls and ≥ 1 egg	21	28,278	1.00	1.00	91	58,032	1.00	1.00
Amount of meat < the size of 2 ping-pong balls and < 1 egg	0	4,063	NA	NA	16	8,684	1.15 (0.67–1.95)	1.18 (0.69–2.02)
**Seafood ≥ 3 times/week**								
No	13	21,067	1.00	1.00	70	21,841	1.00	1.00
Yes	10	12,395	1.48 (0.65–3.41)	1.52 (0.65–3.52)	40	46,798	1.32 (0.90–1.96)	1.30 (0.88–1.93)
**Tofu or soy milk ≥ 3 times/week**								
No	12	19,495	1.00	1.00	64	37,739	1.00	1.00
Yes	11	14,013	1.33 (0.58–3.01)	1.28 (0.56–2.93)	46	30,871	0.90 (0.62–1.32)	0.89 (0.61–1.31)
**Vegetables, seaweed, mushrooms (except kimchi) every meal**								
No	8	17,637	1.00	1.00	51	33,156	1.00	1.00
Yes	15	15,918	2.25 (0.95–5.32)	2.13 (0.90–5.06)	59	35,431	1.09 (0.75–1.59)	1.10 (0.75–1.60)
**Fruits ≥ 5 days/week**								
No	14	19,484	1.00	1.00	51	27,171	1.00	1.00
Yes	9	14,007	0.95 (0.41–2.22)	0.89 (0.38–2.08)	57	41,483	0.74 (0.51–1.08)	0.71 (0.49–1.05)
**Milk or dairy products ≥ 5 days/week**								
No	18	22,677	1.00	1.00	78	41,141	1.00	1.00
Yes	5	10,784	0.58 (0.21–1.56)	0.55 (0.20–1.48)	30	27,390	**0.59 (0.39**–**0.90)**	**0.60 (0.39**–**0.92)**
**≥ 3 side dishes (except soup or kimchi) at every meal**								
No	3	6,212	1.00	1.00	23	16,313	1.00	1.00
Yes	20	27,276	1.96 (0.58–6.66)	1.99 (0.58–6.82)	85	52,386	1.25 (0.79–1.98)	1.23 (0.78–1.96)
**Taste salty when eating out**								
No	9	16,075	1.00	1.00	45	25,413	1.00	1.00
Yes	14	17,281	1.28 (0.55–2.96)	1.28 (0.55–2.98)	61	42,901	0.76 (0.52–1.12)	0.77 (0.52–1.13)
**Tend to eat salty food**								
Yes	4	4,517	1.00	1.00	8	5,816	1.00	1.00
Neutral (Medium)	16	22,844	0.73 (0.24–2.18)	0.73 (0.24–2.18)	79	47,564	1.15 (0.56–2.39)	1.14 (0.55–2.35)
No	3	6,146	0.52 (0.12–2.34)	0.51 (0.11–2.29)	21	15,273	0.96 (0.43–2.17)	0.97 (0.43–2.20)
**Grilled meat frequency**								
Never	12	15,795	1.00	1.00	77	42,096	1.00	1.00
Sometimes	11	14,481	0.98 (0.43–2.23)	0.97 (0.42–2.23)	27	22,091	0.65 (0.42–1.01)	0.65 (0.42–1.02)
Often	0	2,753	NA	NA	3	3,166	0.53 (0.17–1.69)	0.54 (0.17–1.71)
N/A (Not applicable)	0	393	NA	NA	1	1,221	0.48 (0.07–3.46)	0.51 (0.07–3.70)

Model 1 was adjusted for age and sex.

Model 2 was adjusted for age, sex, BMI, household income, and occupation.

We additionally analyzed the association between dietary habits and TC risk after stratification by age and sex in Supplementary Table 3 and stratification by sex and smoking status in Supplementary Table 4. A decreased TC risk was found among those who had a meal duration greater than 10 min in women older than 50 years (aHR, 0.47; 95% CI, 0.28–0.78) and women who never smoked (aHR, 0.63; 95% CI, 0.42–0.94). Similarly, consuming milk and/or dairy products five or more days per week may be inversely associated with TC risk for these small subgroups, with aHRs of 0.39 (95% CI, 0.22–0.71) for older women and 0.54 (0.35–0.85) for women who never smoked.

### Sensitivity analysis

In the sensitivity analysis, using a matched population, the association between dietary habits and TC risk is shown in Supplementary Table 5. The results in the matched population were similar to those in the total population. A significantly reduced TC risk was also found in participants who had a meal duration longer than 10 min (aHR, 0.66; 95% CI, 0.46–0.95) or consumed milk and/or dairy products more than 5 days per week (aHR, 0.65; 95% CI, 0.44–0.97). In addition, these inverse relationships appeared only among individuals aged ≥ 50 years, with aHRs of 0.46 (95% CI, 0.28–0.76) and 0.45 (95% CI, 0.26–0.79), respectively.

## Discussion

This study assessed the relationship between some dietary habits and TC development in a relatively large prospective cohort in South Korea. Among the specific dietary habits, after adjustment for confounders, dairy consumption five or more days a week and meal durations longer than 10 min were found to be two protective factors against TC, especially among participants older than 50 years, women and those who never smoked.

Diet may affect TC development. This relationship can be assessed in several dietary aspects, such as nutrient intake and food consumption frequency and etc. In addition, previous findings suggest that the risk of TC is also modified by dietary habits (9). It is evident that a single nutrient or food intake may influence the cancer risk while it limits to assess the overall eating habits. However, the evaluation of the association between dietary behaviors and cancer risk could suggest a wider and more practical estimation of the relationship between eating habits and cancer risk (31). Therefore, dietary behaviors might contribute greatly to the prevention of cancer (32), including TC. In addition, since this study was carried out with specific dietary habit questionnaires in the Korean population, our findings provide evidence of the impact of eating habits on TC risk for these inhabitants. However, the results of our study may differ from those attained in other populations, such as Caucasian and Hispanic populations, due to differences in dietary patterns and lifestyle factors.

The evidence of an association between TC and dairy consumption in the Asian population is still limited. To the best of our knowledge, only one Japanese cohort study has investigated the relationship between TC risk and dairy consumption; they found reduced risk of TC in those who consumed higher amounts of dairy products, which is consistent with our findings (33). However, the impact of dairy consumption on the development of TC had inconsistent results in other geographical regions. A case-control study in Italy indicated that subjects with a higher dairy intake had a lower risk of TC (9). Conversely, another case-control study in Sweden and Norway reported that high intakes of butter and cheese were risk factors for TC development (34). Moreover, no significant relationship between dairy consumption and TC risk was found in other case-control studies (35, 36).

Biological mechanisms that could explain the protective role of dairy products in reducing TC risk have been proposed. Milk and dairy products are known as an essential source of many important nutrients, including calcium, liposoluble vitamins (A, D, E, and K), and essential fatty acids (37). Dairy fatty acids, particularly butyric acid and conjugated linolenic acid (CLnA), might be anticancer molecules with bioactivity that is mediated by the downregulation of acetyl-CoA carboxylase (ACC), fatty acid synthase (FASN), and 3-hydroxy-3-methylglutaryl coenzyme-A (HMG-CoA) or specific genes related to cell proliferation and apoptosis (38). CLnA was shown to be metabolized to rumenic acid (39), which has a possible beneficial effect on cancer and immunology (38). In addition, linolenic acid may be synthesized endogenously to docosahexaenoic acid (DHA), a long-chain fatty acid found to be inversely associated with TC risk (40). In addition, milk products contain vitamin D (41), and the type of vitamin might have a protective function against tumors (42). After absorption, this vitamin undergoes metabolism in the liver and kidney to become a biologically active metabolite (43). This metabolized compound can strongly bind to the vitamin D receptor on the surface of TC cells and consequently block the TC development stage *via* inhibition of cell growth (44).

In addition, a meal duration of more than 10 min was found to reduce the TC risk in our study population. To our knowledge, there have been no studies on the direct relationship between meal duration and TC development to date. The speed of eating might be considered a factor affecting the length of meal duration. Several previous studies found that eating fast could increase the risk of obesity and metabolic syndrome (45, 46) due to a reduction in insulin sensitivity (47). Otsuka et al. indicated that rapid speed of eating could trigger overeating before the stomach sensation of fullness because of a lack of satiety (46). Although there was no significant difference in BMI between individuals with and without TC in our study due to the relatively small number of individuals with incident TC and the lack of follow-up regarding anthropometric changes, previous studies reported positive associations among obesity, metabolic syndrome and TC (48, 49). Hyperinsulinemia due to obesity causes excessive insulin binding to IGF-1 receptors in the thyroid, increasing the proliferation of TC cells (50). Moreover, the mitogen-activated protein kinase (MAPK) pathway is triggered by the presence of insulin, which induces the dedifferentiation and proliferation of thyroid follicular cells (49).

TC development is believed to have a long latency period (51), and food habits from the initiation of latent disease to the diagnosis of TC could be important. Even though eating habits are relatively stable, alterations in dietary habits could occur and vary among participants over a long follow-up period (52). Dietary habits may be altered due to changes in food availability or active decisions leading to changes in dietary and lifestyle behaviors (53). Therefore, investigation of dietary habit changes during the follow-up period is necessary. However, our study examined eating habits only at baseline. In addition, there were clearly strong risk factors that might have impacted TC risk throughout the follow-up period. Previous findings indicated that ionizing radiation and xenobiotic exposure have clear positive associations with TC risk (11); these factors, combined with dietary habits, could influence the development of TC. However, we did not examine them comprehensively in this study.

In addition, during the follow-up period, a much higher proportion of women had incident TC compared to men in our study (Table 1), which is consistent with many previous findings (54, 55). Women have a higher risk for TC development due to the influence of sex hormones (56). During the reproductive period, women are influenced by estrogen, which is a strong growth factor for TC cells. High levels of estrogen in women could activate the estrogen receptor linked to the tyrosine kinase signaling pathways MAPK and PI3K for TC cell survival and invasion and TC progression (57). Herein, we additionally evaluated the association between sex and TC risk (Supplementary Table 6), and a significantly increased TC risk was observed among women (aHR, 2.95; 95% CI, 1.49–5.84). In contrast, we found no significant relationship between smoking status and TC development in the study population. Moreover, there was a low percentage of women who smoked in our study (7.40%). Thus, the association of dietary habits with TC could be impacted by women more than by smoking status. However, several previous studies indicated that smoking may decrease the risk of TC (20). Therefore, further studies are needed to confirm this association.

Healthy dietary habits play critical roles in the prevention of cancer, including TC. Thus, it is necessary to study and identify healthy eating habit recommendations. Our study indicates that dairy consumption five or more days a week and meal durations longer than 10 min could reduce TC development, which may contribute to the improvement of diet behaviors for TC prevention in the Korean population. Moreover, dairy foods are known as healthy food and their consumption has been suggested to be inversely associated with several cancer types and metabolic syndrome in previous Korean studies (58, 59). Therefore, high dairy consumption is a potential healthy lifestyle factor that decreases the risk of TC. To our knowledge, this study also presents the first investigation of the relationship between meal duration and TC development. In addition to eating speed, meal duration may also be related to food types, work traits, and other lifestyle-related factors. The findings in our study support the opinion that meal duration may be the dietary behavior marker, which is a helpful predictor of a healthy lifestyle for TC prevention.

There are several strengths of our study. To the best of our knowledge, this is the first prospective cohort study in South Korea to evaluate the relationship between some aspects of dietary habits and TC incidence. This prospective cohort study had a large sample size with a relatively long median follow-up period of 7.6 years. Moreover, we collected dietary information from study participants at enrollment; thus, selection or recall bias was prevented. In addition, analyses were adjusted for demographic information to control the impact of confounding factors. Additionally, assuming that bias may be associated with the large difference between incident TC cases and non-incident cases, we carried out sensitivity analysis using a population matched by a 1:2 ratio of TC cases and non-cases based on the propensity score matching method. However, we found that the results were consistent in both main and the matched population analyses.

Our study still has several limitations. First, we evaluated the diet information only at baseline and did not investigate whether dietary habits changed over the follow-up period. Second, our data had many missing values, as reported in Table 1. However, we excluded the missing values for the association analysis to minimize their impact on the results. Third, the dietary habit questions were only related to consumption frequency and did not include questions on dietary intake in which an objective indication of diet evaluation might be limited. Accordingly, we could not calculate the intake of each food and separate the study populations into subgroups by food intake. Fourth, the dietary habit questionnaire consisted of only 12 items assessing dietary factors, which might not comprehensively reflect the effect of all food items on the development of TC. However, we tried to formulate questions that represented the foods, dietary patterns, and eating habits specific to the Korean population. Fifth, there were several environmental exposures and nutritional factors (e.g., history of benign thyroid nodules/adenoma, ionizing radiation, and iodine intake) that were not measured completely in the subjects in the present study. These factors may result in bias in investigations of the relationship. Moreover, specific types of TC based on histology, e.g., papillary TC, should be examined in further studies.

## Conclusion

In conclusion, our results imply that consuming milk and/or dairy products 5 or more days a week and meal durations greater than 10 min could be protective factors against TC in the population, exerting a stronger protective effect in individuals above age of 50 years, women, and those who never smoked. Further prospective studies with longer follow-up periods are needed to investigate dietary intake with more food items, including dietary patterns with respect to specific types of TC, while accounting for additional nutritional and environmental factors that could affect the results.

## Data availability statement

The original contributions presented in this study are included in this article/Supplementary material, further inquiries can be directed to the corresponding author.

## Ethics statement

The studies involving human participants were reviewed and approved by the Institution Review Board of NCC, Korea (No. NCCNCS-07-077). The patients/participants provided their written informed consent to participate in this study.

## Author contributions

LN designed and conducted the research, analyzed the data, and wrote the manuscript draft. MG revised the manuscript. JL collected and analyzed the data. JK designed and conducted the research, collected the data, and revised the manuscript. All authors read and approved the final manuscript.
