# Supplementary material for: Association between dietary habits and incident thyroid cancer: A prospective cohort study
Source: Front Nutr. 2023 Feb 15;10:1104925. doi: 10.3389/fnut.2023.1104925 (PMC9975340; doi:10.3389/fnut.2023.1104925)
Supplement: Supplementary file 1 [file Table_1.DOCX]

**Supplementary Table S1.** Baseline characteristics of the study participants in matched population (1:2)

| **Characteristic** | **Total** | | | **Age group** | | | | | |
| --- | --- | --- | --- | --- | --- | --- | --- | --- | --- |
|  |  |  |  | **< 50 years** | | | **≥ 50 years** | | |
|  | ***Nonincident cases (n=276)*** | ***Incident TC cases (n=138)*** | ***p-value^a^*** | ***Nonincident cases (n=124)*** | ***Incident TC cases (n=62)*** | ***p-value^a^*** | ***Nonincident cases (n=152)*** | ***Incident TC cases (n=76)*** | ***p-value^a^*** |
| ***Age, mean ± SD, years*** | 50.9 ± 7.6 | 50.9 ± 7.7 | 0.999 | 44.1 ± 4.1 | 44.1 ± 4.1 | 0.999 | 56.4 ± 4.9 | 56.4 ± 4.9 | 0.999 |
| ***Sex (n, %)*** |  |  |  |  |  |  |  |  |  |
| Men | 36 (13.0) | 18 (13.0) | 0.999 | 12 (9.7) | 6 (9.7) | 0.999 | 24 (15.8) | 12 (15.8) | 0.999 |
| Women | 240 (87.0) | 120 (87.0) |  | 112 (90.3) | 56 (90.3) |  | 128 (84.2) | 64 (84.2) |  |
| ***BMI*** (kg/m²) |  |  |  |  |  |  |  |  |  |
| <23 | 132 (47.8) | 60 (43.5) | 0.806 | 76 (61.3) | 31 (50.0) | 0.391 | 56 (36.8) | 29 (38.2) | 0.877 |
| 23 -<25 | 68 (24.6) | 34 (24.6) |  | 24 (19.4) | 15 (24.2) |  | 44 (29.0) | 19 (25.0) |  |
| ≥25 | 71 (25.7) | 38 (27.5) |  | 22 (17.7) | 14 (22.6) |  | 49 (32.2) | 24 (31.6) |  |
| Missing | 5 (1.8) | 6 (4.4) |  | 2 (1.6) | 2 (3.2) |  | 3 (2.0) | 4 (5.3) |  |
| ***Smoking status*** |  |  |  |  |  |  |  |  |  |
| Current smoker | 21 (7.6) | 10 (7.3) | 0.926 | 11 (8.9) | 6 (9.7) | 0.669 | 10 (6.6) | 4 (5.3) | 0.550 |
| Ex-smoker | 28 (10.1) | 13 (9.4) |  | 6 (4.8) | 5 (8.1) |  | 22 (14.5) | 8 (10.5) |  |
| Never smoker | 218 (79.0) | 114 (82.6) |  | 104 (83.4) | 50 (80.7) |  | 114 (75.0) | 64 (84.2) |  |
| Missing | 9 (3.3) | 1 (0.7) |  | 3 (2.4) | 1 (1.6) |  | 6 (4.0) | 0 (0.0) |  |
| ***Alcohol consumption*** |  |  |  |  |  |  |  |  |  |
| Current drinker | 138 (50.0) | 67 (48.6) | 0.308 | 67 (54.0) | 37 (59.7) | 0.515 | 71 (46.7) | 30 (39.5) | 0.278 |
| Ex-drinker | 18 (6.5) | 5 (3.6) |  | 6 (4.8) | 1 (1.6) |  | 12 (7.9) | 4 (5.3) |  |
| Never drinker | 113 (40.9) | 66 (47.8) |  | 47 (37.9) | 24 (38.7) |  | 66 (43.4) | 42 (55.3) |  |
| Missing | 7 (2.5) | 0 (0.0) |  | 4 (3.2) | 0 (0.0) |  | 3 (2.0) | 0 (0.0) |  |
| ***Regular exercise*** |  |  |  |  |  |  |  |  |  |
| Yes | 2 (0.7) | 54 (39.1) | 0.733 | 1 (0.8) | 20 (32.3) | 0.534 | 1 (0.7) | 34 (44.7) | 0.351 |
| No | 3 (1.1) | 59 (42.8) |  | 3 (2.4) | 29 (46.8) |  | 0 (0.0) | 30 (39.5) |  |
| Missing | 271 (98.2) | 25 (18.1) |  | 120 (96.8) | 13 (21.0) |  | 151 (99.3) | 12(15.8) |  |
| ***First-degree family history of TC*** |  |  |  |  |  |  |  |  |  |
| Yes | 3 (1.1) | 6 (4.4) | **0.034** | 2 (1.6) | 2 (3.2) | 0.480 | 1 (0.7) | 4 (5.3) | **0.027** |
| No | 268 (97.1) | 131 (94.9) |  | 119 (96.0) | 59 (95.2) |  | 149 (98.0) | 74 (94.7) |  |
| Missing | 5 (1.8) | 1 (0.7) |  | 3 (2.4) | 1 (1.6) |  | 2 (1.3) | 0 (0.0) |  |
| ***Marital status*** |  |  |  |  |  |  |  |  |  |
| Married | 225 (81.5) | 113 (81.9) | 0.621 | 103 (83.1) | 52 (83.9) | 0.915 | 122 (80.3) | 61 (80.3) | 0.475 |
| Other | 46 (16.7) | 20 (14.5) |  | 17 (13.7) | 9 (14.5) |  | 29 (19.1) | 11 (14.5) |  |
| Missing | 5 (1.8) | 5 (3.6) |  | 4 (3.2) | 1 (1.6) |  | 1 (0.7) | 4 (5.3) |  |
| ***Household Income,*** in 10,000 Korean won/month |  |  |  |  |  |  |  |  |  |
| < 200 | 60 (21.7) | 17 (12.3) | **0.008** | 21 (16.9) | 9 (14.5) | 0.064 | 39 (25.7) | 8 (10.5) | **0.032** |
| 200-400 | 97 (35.1) | 45 (32.6) |  | 41 (33.1) | 14 (22.6) |  | 56 (36.8) | 31 (40.8) |  |
| ≥ 400 | 69 (25.0) | 52 (37.7) |  | 41 (33.1) | 33 (53.2) |  | 28 (18.4) | 19 (25.0) |  |
| Missing | 50 (18.1) | 24 (17.4) |  | 21 (16.9) | 6 (9.7) |  | 29 (19.1) | 18 (23.7) |  |
| ***Educational level*** |  |  |  |  |  |  |  |  |  |
| Middle school | 65 (23.6) | 15 (10.9) | **0.023** | 16 (12.9) | 2 (3.2) | 0.097 | 49 (32.2) | 13 (17.1) | 0.156 |
| High school | 95 (34.4) | 52 (37.7) |  | 39 (31.5) | 24 (38.7) |  | 56 (36.8) | 28 (36.8) |  |
| College or more | 110 (39.9) | 57 (41.3) |  | 66 (53.2) | 33 (53.2) |  | 44 (29.0) | 24 (31.6) |  |
| Missing | 6 (2.2) | 14 (10.1) |  | 3 (2.4) | 3 (4.8) |  | 3 (2.0) | 11 (14.5) |  |
| ***Occupation*** |  |  |  |  |  |  |  |  |  |
| Group 1: Professionals and administrative  management | 45 (16.3) | 18 (13.0) | **0.014** | 30 (24.2) | 11 (17.7) | 0.362 | 15 (9.9) | 7 (9.2) | **0.029** |
| Group 2: Office, sales, and service positions | 33 (12.0) | 33 (23.9) |  | 20 (16.1) | 16 (25.8) |  | 13 (8.6) | 17 (22.4) |  |
| Group 3: Agriculture and laborers | 13 (4.7) | 4 (2.9) |  | 4 (3.2) | 1 (1.6) |  | 9 (5.9) | 3 (4.0) |  |
| Group 4: Unemployed or others | 178 (64.5) | 78 (56.5) |  | 66 (53.2) | 32 (51.6) |  | 112 (73.7) | 46 (60.5) |  |
| Missing | 7 (2.5) | 5 (3.6) |  | 4 (3.2) | 2 (3.2) |  | 3 (2.0) | 3 (4.0) |  |

^a^χ^2^ tests and t tests were used for categorical variables and continuous variables, respectively.

**Supplementary Table S2.** Difference of estrogen-related characteristics of the women participants in the study

| **Characteristic** | ***Nonincident cases (n=8931)*** | ***Incident TC cases (n=120)*** | ***p-value^a^*** |
| --- | --- | --- | --- |
| ***Menarche age, mean ± SD, years*** | 14.73 ± 1.76 | 14.47 ± 1.62 | 0.108 |
| ***Menopausal status*** |  |  |  |
| Yes | 3059 (34.3) | 50 (41.7) | 0.079 |
| No | 5382 (60.3) | 63 (52.5) |  |
| Missing | 490 (5.5) | 7 (5.8) |  |

^a^χ^2^ tests and t tests were used for categorical variables and continuous variables, respectively.

**Supplementary Table S3.** HRs and 95% CIs of incident TC related to dietary habits by stratification of age and sex

| **Dietary habit** | **Age group** | | | | | | | | | | | | | | | |
| --- | --- | --- | --- | --- | --- | --- | --- | --- | --- | --- | --- | --- | --- | --- | --- | --- |
|  | **< 50 years** | | | | | | | | **≥ 50 years** | | | | | | | |
|  | **Men** | | | | **Women** | | | | **Men** | | | | **Women** | | | |
|  | ***TC cases*** | ***Person-years*** | ***HR (95% CI)  for Model 1*** | ***HR (95% CI)  for Model 2*** | ***TC cases*** | ***Person-years*** | ***HR (95% CI)  for Model 1*** | ***HR (95% CI)  for Model 2*** | ***TC cases*** | ***Person-years*** | ***HR (95% CI)  for Model 1*** | ***HR (95% CI)  for Model 2*** | ***TC cases*** | ***Person-years*** | ***HR (95% CI)  for Model 1*** | ***HR (95% CI)  for Model 2*** |
| ***Meal frequency: 3 meals/day for ≥ 5 days/week*** | | | | | | | | | | | | | | | | |
| No | 2 | 2,802 | 1.00 | 1.00 | 15 | 8,427 | 1.00 | 1.00 | 0 | 3,320 | 1.00 | 1.00 | 13 | 9,354 | 1.00 | 1.00 |
| Yes | 4 | 7,773 | 0.69 (0.13-3.79) | 0.65 (0.11-3.83) | 39 | 17,705 | 1.29 (0.71-2.34) | 1.30 (0.70-2.39) | 12 | 22,398 | NA | NA | 49 | 30,563 | 1.18 (0.64-2.17) | 1.25 (0.67-2.34) |
| ***Meal duration ≥ 10 minutes*** | | | | | | | | | | | | | | | | |
| No | 4 | 2,867 | 1.00 | 1.00 | 13 | 5,519 | 1.00 | 1.00 | 4 | 6,406 | 1.00 | 1.00 | 25 | 9,543 | 1.00 | 1.00 |
| Yes | 2 | 7,716 | 0.19 (0.03-1.01) | 0.19 (0.04-1.08) | 41 | 20,618 | 0.84 (0.45-1.57) | 0.81 (0.43-1.52) | 8 | 19,304 | 0.66 (0.20-2.21) | 0.58 (0.18-1.96) | 37 | 30,386 | **0.47 (0.28-0.77)** | **0.47 (0.28-0.78)** |
| ***Meat and eggs ≥ 5 times/week*** | | | | | | | | | | | | | | | | |
| No meat or amount of meat ≥ the size of 2 ping-pong balls and ≥ 1 egg | 5 | 9,017 | 1.00 | 1.00 | 46 | 22,102 | 1.00 | 1.00 | 10 | 21,625 | 1.00 | 1.00 | 52 | 33,690 | 1.00 | 1.00 |
| Amount of meat < the size of 2 ping-pong balls and < 1 egg | 0 | 1,202 | NA | NA | 7 | 3,421 | 0.98 (0.44-2.17) | 0.95 (0.43-2.12) | 1 | 3,151 | 0.66 (0.09-5.17) | 0.68 (0.09-5.31) | 8 | 5,010 | 1.03 (0.49-2.16) | 1.07 (0.51-2.25) |
| ***Seafood ≥ 3 times/week*** | | | | | | | | | | | | | | | | |
| No | 2 | 6,810 | 1.00 | 1.00 | 36 | 18,497 | 1.00 | 1.00 | 8 | 15,640 | 1.00 | 1.00 | 38 | 26,960 | 1.00 | 1.00 |
| Yes | 4 | 3,773 | 3.39 (0.62-18.59) | 3.44 (0.59-20.10) | 18 | 7,615 | 1.29 (0.73-2.27) | 1.28 (0.73-2.27) | 4 | 9,990 | 0.80 (0.24-2.66) | 0.72 (0.21-2.42) | 24 | 12,978 | 1.35 (0.81-2.25) | 1.32 (0.79-2.21) |
| ***Tofu or soy milk ≥ 3 times/week*** | | | | | | | | | | | | | | | | |
| No | 3 | 6,611 | 1.00 | 1.00 | 37 | 15,338 | 1.00 | 1.00 | 6 | 14,424 | 1.00 | 1.00 | 31 | 20,919 | 1.00 | 1.00 |
| Yes | 3 | 3,972 | 1.61 (0.32-7.98) | 1.42 (0.28-7.21) | 17 | 10,771 | 0.67 (0.37-1.18) | 0.69 (0.38-1.21) | 6 | 11,234 | 1.30 (0.42-4.03) | 1.12 (0.36-3.51) | 31 | 19,011 | 1.11 (0.67-1.82) | 1.11 (0.67-1.83) |
| ***Vegetables, seaweed, mushrooms (except kimchi) every meal*** | | | | | | | | | | | | | | | | |
| No | 2 | 5,642 | 1.00 | 1.00 | 26 | 12,788 | 1.00 | 1.00 | 4 | 12,748 | 1.00 | 1.00 | 28 | 19,718 | 1.00 | 1.00 |
| Yes | 4 | 4,950 | 2.22 (0.41-12.13) | 1.97 (0.34-11.29) | 28 | 13,335 | 1.04 (0.61-1.77) | 1.04 (0.61-1.79) | 8 | 12,941 | 1.98 (0.60-6.58) | 1.80 (0.54-6.05) | 34 | 20,182 | 1.18 (0.72-1.95) | 1.19 (0.72-1.97) |
| ***Fruits ≥ 5 days/week*** | | | | | | | | | | | | | | | | |
| No | 3 | 6,961 | 1.00 | 1.00 | 28 | 11,737 | 1.00 | 1.00 | 6 | 13,251 | 1.00 | 1.00 | 28 | 14,757 | 1.00 | 1.00 |
| Yes | 3 | 3,621 | 1.89 (0.38-9.39) | 1.76 (0.35-8.91) | 25 | 14,371 | 0.75 (0.44-1.28) | 0.68 (0.39-1.19) | 6 | 12,406 | 1.08 (0.35-3.34) | 0.82 (0.26-2.63) | 33 | 25,203 | 0.69 (0.42-1.15) | 0.68 (0.41-1.13) |
| ***Milk or Dairy products ≥ 5 days/week*** | | | | | | | | | | | | | | | | |
| No | 4 | 7,639 | 1.00 | 1.00 | 38 | 17,156 | 1.00 | 1.00 | 8 | 16,744 | 1.00 | 1.00 | 47 | 22,339 | 1.00 | 1.00 |
| Yes | 2 | 2,936 | 1.29 (0.24-7.05) | 1.30 (0.24-7.23) | 15 | 8,934 | 0.77 (0.42-1.39) | 0.76 (0.42-1.38) | 4 | 8,862 | 0.94 (0.28-3.14) | 0.81 (0.24-2.73) | 14 | 17,543 | **0.38 (0.21-0.69)** | **0.39 (0.22-0.71)** |
| ***≥ 3 side dishes (except soup or kimchi) at every meal*** | | | | | | | | | | | | | | | | |
| No | 1 | 1,835 | 1.00 | 1.00 | 10 | 7,498 | 1.00 | 1.00 | 2 | 3,975 | 1.00 | 1.00 | 13 | 9,278 | 1.00 | 1.00 |
| Yes | 5 | 8,734 | 1.01 (0.12-8.66) | 1.11 (0.12-10.10) | 43 | 18,633 | 1.74 (0.88-3.47) | 1.73 (0.86-3.45) | 10 | 21,704 | 0.92 (0.20-4.21) | 0.86 (0.19-3.94) | 48 | 30,692 | 1.12 (0.61-2.07) | 1.12 (0.61-2.08) |
| ***Taste salty when eating out*** | | | | | | | | | | | | | | | | |
| No | 4 | 4,914 | 1.00 | 1.00 | 20 | 9,073 | 1.00 | 1.00 | 5 | 12,239 | 1.00 | 1.00 | 25 | 15,356 | 1.00 | 1.00 |
| Yes | 2 | 5,645 | 0.43 (0.08-2.37) | 0.47 (0.08-2.67) | 33 | 17,000 | 0.85 (0.49-1.49) | 0.81 (0.46-1.43) | 7 | 13,301 | 1.26 (0.40-3.99) | 1.13 (0.35-3.61) | 34 | 24,303 | 0.84 (0.50-1.41) | 0.80 (0.47-1.35) |
| ***Tend to eat salty food*** | | | | | | | | | | | | | | | | |
| Yes | 1 | 1,429 | 1.00 | 1.00 | 2 | 2,057 | 1.00 | 1.00 | 2 | 3,313 | 1.00 | 1.00 | 7 | 3,558 | 1.00 | 1.00 |
| Neutral (Medium) | 5 | 7,215 | 1.00 (0.12-8.59) | 0.89 (0.10-7.74) | 39 | 18,519 | 2.13 (0.51-8.82) | 2.09 (0.50-8.67) | 8 | 17,155 | 0.76 (0.16-3.59) | 0.74 (0.16-3.49) | 44 | 27,641 | 0.80 (0.36-1.78) | 0.80 (0.36-1.79) |
| No | 0 | 1,948 | NA | NA | 12 | 5,555 | 2.18 (0.49-9.72) | 2.25 (0.50-10.13) | 2 | 5,197 | 0.63 (0.09-4.47) | 0.57 (0.08-4.12) | 10 | 8,734 | 0.57 (0.22-1.51) | 0.56 (0.21-1.49) |

**Supplementary Table S3.** *Cont.*

| **Dietary habit** | **Age group** | | | | | | | | | | | | | | | |
| --- | --- | --- | --- | --- | --- | --- | --- | --- | --- | --- | --- | --- | --- | --- | --- | --- |
|  | **< 50 years** | | | | | | | | **≥ 50 years** | | | | | | | |
|  | **Men** | | | | **Women** | | | | **Men** | | | | **Women** | | | |
|  | ***TC cases*** | ***Person-years*** | ***HR (95% CI)  for Model 1*** | ***HR (95% CI)  for Model 2*** | ***TC cases*** | ***Person-years*** | ***HR (95% CI)  for Model 1*** | ***HR (95% CI)  for Model 2*** | ***TC cases*** | ***Person-years*** | ***HR (95% CI)  for Model 1*** | ***HR (95% CI)  for Model 2*** | ***TC cases*** | ***Person-years*** | ***HR (95% CI)  for Model 1*** | ***HR (95% CI)  for Model 2*** |
| ***Grilled meat frequency*** | | | | | | | | | | | | | | | | |
| Never | 1 | 4,310 | 1.00 | 1.00 | 38 | 14,125 | 1.00 | 1.00 | 10 | 12,908 | 1.00 | 1.00 | 41 | 26,662 | 1.00 | 1.00 |
| Sometimes | 5 | 5,112 | 4.23 (0.49-36.17) | 4.16 (0.47-36.54) | 14 | 10,456 | **0.49 (0.27-0.91)** | **0.48 (0.26-0.88)** | 2 | 10,344 | 0.25 (0.06-1.15) | 0.26 (0.06-1.19) | 17 | 10,710 | 1.04 (0.59-1.82) | 1.03 (0.58-1.81) |
| Often | 0 | 1,051 | NA | NA | 1 | 1,268 | 0.30 (0.04-2.15) | 0.29 (0.04-2.10) | 0 | 1,991 | NA | NA | 2 | 1,608 | 0.81 (0.20-3.36) | 0.82 (0.20-3.39) |
| N/A (Not applicable) | 0 | 75 | NA | NA | 0 | 254 | NA | NA | 0 | 359 | NA | NA | 1 | 927 | 0.70 (0.10-5.11) | 0.79 (0.11-0.74) |

Model 1 was crude model.

Model 2 was adjusted for BMI, smoking status, household income and occupation; and additionally adjusted for menarche age, menopausal status in women.

**Supplementary Table S4.** HRs and 95% CIs of incident TC related to dietary habits by stratification of sex and smoking status

| **Dietary habit** | **Men** | | | | | | | | **Women** | | | | | | | |
| --- | --- | --- | --- | --- | --- | --- | --- | --- | --- | --- | --- | --- | --- | --- | --- | --- |
|  | **Ever smoker** | | | | **Never smoker** | | | | **Ever smoker** | | | | **Never smoker** | | | |
|  | ***TC cases*** | ***Person-years*** | ***HR (95% CI)  for Model 1*** | ***HR (95% CI)  for Model 2*** | ***TC cases*** | ***Person-years*** | ***HR (95% CI)  for Model 1*** | ***HR (95% CI)  for Model 2*** | ***TC cases*** | ***Person-years*** | ***HR (95% CI)  for Model 1*** | ***HR (95% CI)  for Model 2*** | ***TC cases*** | ***Person-years*** | ***HR (95% CI)  for Model 1*** | ***HR (95% CI)  for Model 2*** |
| ***Meal frequency: 3 meals/day for ≥ 5 days/week*** | | | | | | | | | | | | | | | | |
| No | 2 | 5,245 | 1.00 | 1.00 | 0 | 876 | 1.00 | 1.00 | 5 | 2,332 | 1.00 | 1.00 | 23 | 15,425 | 1.00 | 1.00 |
| Yes | 12 | 23,580 | 1.46 (0.32-6.73) | 1.48 (0.32-6.87) | 4 | 6,591 | NA | NA | 4 | 2,387 | 0.83 (0.22-3.10) | 0.66 (0.17-2.60) | 83 | 45,744 | 1.32 (0.83-2.10) | 1.33 (0.84-2.13) |
| ***Meal duration ≥ 10 minutes*** | | | | | | | | | | | | | | | | |
| No | 6 | 7,457 | 1.00 | 1.00 | 2 | 1,817 | 1.00 | 1.00 | 4 | 1,152 | 1.00 | 1.00 | 34 | 13,873 | 1.00 | 1.00 |
| Yes | 8 | 21,358 | 0.48 (0.16-1.37) | 0.43 (0.15-1.24) | 2 | 5,662 | 0.34 (0.05-2.38) | 0.32 (0.04-2.63) | 5 | 3,558 | 0.40 (0.11-1.48) | 0.30 (0.08-1.20) | 72 | 47,320 | **0.62 (0.41-0.93)** | **0.63 (0.42-0.94)** |
| ***Meat and eggs ≥ 5 times/week*** | | | | | | | | | | | | | | | | |
| No meat or amount of meat ≥ the size of 2 ping-pong balls and ≥ 1 egg | 12 | 24,311 | 1.00 | 1.00 | 3 | 6,331 | 1.00 | 1.00 | 9 | 3,967 | 1.00 | 1.00 | 88 | 51,700 | 1.00 | 1.00 |
| Amount of meat < the size of 2 ping-pong balls and < 1 egg | 0 | 3,493 | NA | NA | 1 | 860 | 2.40 (0.25-23.05) | 2.15 (0.20-23.77) | 0 | 570 | NA | NA | 15 | 7,824 | 1.10 (0.64-1.92) | 1.14 (0.66-1.98) |
| ***Seafood ≥ 3 times/week*** | | | | | | | | | | | | | | | | |
| No | 7 | 17,726 | 1.00 | 1.00 | 3 | 4,724 | 1.00 | 1.00 | 6 | 3,341 | 1.00 | 1.00 | 67 | 42,074 | 1.00 | 1.00 |
| Yes | 7 | 11,025 | 1.64 (0.58-4.68) | 1.70 (0.58-4.95) | 1 | 2,738 | 0.58 (0.06-5.66) | 0.26 (0.02-4.08) | 3 | 1,371 | 1.28 (0.32-5.15) | 1.41 (0.34-5.84) | 39 | 19,103 | 1.36 (0.92-2.03) | 1.34 (0.90-2.00) |
| ***Tofu or soy milk ≥ 3 times/week*** | | | | | | | | | | | | | | | | |
| No | 8 | 16,721 | 1.00 | 1.00 | 1 | 4,313 | 1.00 | 1.00 | 4 | 2,774 | 1.00 | 1.00 | 63 | 33,426 | 1.00 | 1.00 |
| Yes | 6 | 12,069 | 1.07 (0.37-3.08) | 1.01 (0.35-2.95) | 3 | 3,137 | 4.22 (0.44-40.91) | 2.63 (0.25-27.49) | 5 | 1,944 | 1.86 (0.50-6.96) | 1.44 (0.38-5.48) | 43 | 27,734 | 0.85 (0.58-1.25) | 0.84 (0.57-1.24) |
| ***Vegetables, seaweed, mushrooms (except kimchi) every meal*** | | | | | | | | | | | | | | | | |
| No | 5 | 14,906 | 1.00 | 1.00 | 1 | 3,485 | 1.00 | 1.00 | 3 | 2,731 | 1.00 | 1.00 | 50 | 29,672 | 1.00 | 1.00 |
| Yes | 9 | 13,930 | 1.97 (0.66-5.87) | 1.77 (0.59-5.34) | 3 | 3,960 | 2.66 (0.28-25.71) | 1.56 (0.14-17.01) | 6 | 1,988 | 2.73 (0.68-10.91) | 2.71 (0.65-11.26) | 56 | 31,471 | 1.06 (0.72-1.55) | 1.06 (0.72-1.55) |
| ***Fruits ≥ 5 days/week*** | | | | | | | | | | | | | | | | |
| No | 8 | 16,763 | 1.00 | 1.00 | 1 | 3,449 | 1.00 | 1.00 | 6 | 2,721 | 1.00 | 1.00 | 50 | 23,722 | 1.00 | 1.00 |
| Yes | 6 | 12,010 | 1.10 (0.38-3.22) | 0.94 (0.32-2.79) | 3 | 4,017 | 3.37 (0.34-33.21) | 1.96 (0.18-21.40) | 3 | 1,997 | 0.72 (0.18-2.91) | 0.56 (0.14-2.34) | 54 | 37,467 | 0.70 (0.48-1.04) | **0.67 (0.45-0.99)** |
| ***Milk or Dairy products ≥ 5 days/week*** | | | | | | | | | | | | | | | | |
| No | 11 | 19,653 | 1.00 | 1.00 | 1 | 4,730 | 1.00 | 1.00 | 7 | 3,024 | 1.00 | 1.00 | 77 | 36,412 | 1.00 | 1.00 |
| Yes | 3 | 9,090 | 0.60 (0.17-2.17) | 0.52 (0.14-1.88) | 3 | 2,708 | 5.18 (0.54-49.90) | 4.11 (0.42-40.35) | 2 | 1,694 | 0.55 (0.11-2.65) | 0.65 (0.13-3.23) | 27 | 24,682 | **0.53 (0.34-0.83)** | **0.54 (0.35-0.85)** |
| ***≥ 3 side dishes (except soup or kimchi) at every meal*** | | | | | | | | | | | | | | | | |
| No | 2 | 4,758 | 1.00 | 1.00 | 1 | 1,053 | 1.00 | 1.00 | 1 | 1,454 | 1.00 | 1.00 | 22 | 15,261 | 1.00 | 1.00 |
| Yes | 12 | 24,022 | 1.22 (0.27-5.45) | 1.20 (0.27-5.40) | 3 | 6,416 | 0.56 (0.06-5.51) | 0.44 (0.04-4.77) | 8 | 3,254 | 3.61 (0.45-28.85) | 4.59 (0.51-41.58) | 82 | 45,970 | 1.28 (0.80-2.05) | 1.27 (0.79-2.05) |
| ***Taste salty when eating out*** | | | | | | | | | | | | | | | | |
| No | 8 | 14,250 | 1.00 | 1.00 | 1 | 2,903 | 1.00 | 1.00 | 1 | 1,825 | 1.00 | 1.00 | 44 | 22,510 | 1.00 | 1.00 |
| Yes | 6 | 14,424 | 0.73 (0.25-2.11) | 0.69 (0.24-1.99) | 3 | 4,522 | 1.99 (0.21-19.16) | 3.13 (0.22-43.85) | 8 | 2,857 | 4.84 (0.60-38.93) | 5.10 (0.59-44.47) | 58 | 38,379 | 0.74 (0.50-1.09) | 0.73 (0.49-1.09) |
| ***Tend to eat salty food*** | | | | | | | | | | | | | | | | |
| Yes | 3 | 3,980 | 1.00 | 1.00 | 0 | 762 | 1.00 | 1.00 | 1 | 537 | 1.00 | 1.00 | 8 | 5,054 | 1.00 | 1.00 |
| Neutral (Medium) | 9 | 19,530 | 0.61 (0.17-2.25) | 0.60 (0.16-2.23) | 4 | 4,840 | NA | NA | 7 | 3,314 | 1.00 (0.12-8.13) | 0.79 (0.09-6.74) | 75 | 42,724 | 1.08 (0.52-2.24) | 1.06 (0.51-2.20) |
| No | 2 | 5,278 | 0.51 (0.09-3.04) | 0.49 (0.08-2.98) | 0 | 1,866 | NA | NA | 1 | 867 | 0.57 (0.04-9.06) | 0.38 (0.02-6.42) | 21 | 13,407 | 0.95 (0.42-2.15) | 0.95 (0.42-2.16) |

**Supplementary Table S4.** *Cont.*

| **Dietary habit** | **Men** | | | | | | | | **Women** | | | | | | | |
| --- | --- | --- | --- | --- | --- | --- | --- | --- | --- | --- | --- | --- | --- | --- | --- | --- |
|  | **Ever smoker** | | | | **Never smoker** | | | | **Ever smoker** | | | | **Never smoker** | | | |
|  | ***TC cases*** | ***Person-years*** | ***HR (95% CI)  for Model 1*** | ***HR (95% CI)  for Model 2*** | ***TC cases*** | ***Person-years*** | ***HR (95% CI)  for Model 1*** | ***HR (95% CI)  for Model 2*** | ***TC cases*** | ***Person-years*** | ***HR (95% CI)  for Model 1*** | ***HR (95% CI)  for Model 2*** | ***TC cases*** | ***Person-years*** | ***HR (95% CI)  for Model 1*** | ***HR (95% CI)  for Model 2*** |
| ***Grilled meat frequency*** | | | | | | | | | | | | | | | | |
| Never | 8 | 13,431 | 1.00 | 1.00 | 3 | 3,787 | 1.00 | 1.00 | 4 | 2,364 | 1.00 | 1.00 | 74 | 38,309 | 1.00 | 1.00 |
| Sometimes | 6 | 12,496 | 0.78 (0.27-2.27) | 0.77 (0.26-2.27) | 1 | 2,959 | 0.35 (0.04-3.43) | 0.16 (0.01-2.68) | 5 | 1,985 | 1.38 (0.37-5.19) | 1.38 (0.35-5.43) | 26 | 19,132 | 0.67 (0.42-1.05) | 0.67 (0.42-1.05) |
| Often | 0 | 2,488 | NA | NA | 0 | 554 | NA | NA | 0 | 264 | NA | NA | 3 | 2,612 | 0.58 (0.18-1.83) | 0.59 (0.19-1.88) |
| N/A (Not applicable) | 0 | 300 | NA | NA | 0 | 134 | NA | NA | 0 | 93 | NA | NA | 1 | 1,087 | 0.50 (0.07-3.59) | 0.54 (0.08-3.91) |

Model 1 was adjusted for age.

Model 2 was adjusted for age, BMI, household income and occupation; and additionally adjusted for menarche age, menopausal status in women.

**Supplementary Table S5.** HRs and 95% CIs of incident TC related to dietary habits by stratification of age in matched population (1:2)

| **Dietary habit** | **Total population** | | | | **Age group** | | | | | | | |
| --- | --- | --- | --- | --- | --- | --- | --- | --- | --- | --- | --- | --- |
|  |  |  |  |  | **< 50 years** | | | | **≥ 50 years** | | | |
|  | ***TC cases*** | ***Person-years*** | ***HR (95% CI)  for Model 1*** | ***HR (95% CI)  for Model 2*** | ***TC cases*** | ***Person-years*** | ***HR (95% CI)  for Model 1*** | ***HR (95% CI)  for Model 2*** | ***TC cases*** | ***Person-years*** | ***HR (95% CI)  for Model 1*** | ***HR (95% CI)  for Model 2*** |
| ***Meal frequency: 3 meals/day for ≥ 5 days/week*** | | | | | | | | | | | | |
| No | 30 | 771 | 1.00 | 1.00 | 17 | 359 | 1.00 | 1.00 | 13 | 413 | 1.00 | 1.00 |
| Yes | 104 | 2,741 | 0.99 (0.66-1.49) | 1.34 (0.88-2.03) | 43 | 1,220 | 0.77 (0.44-1.35) | 0.69 (0.37-1.28) | 61 | 1,521 | 1.26 (0.69-2.31) | 1.29 (0.68-2.45) |
| ***Meal duration ≥ 10mins*** | | | | | | | | | | | | |
| No | 46 | 851 | 1.00 | 1.00 | 17 | 459 | 1.00 | 1.00 | 29 | 392 | 1.00 | 1.00 |
| Yes | 88 | 2,649 | **0.64 (0.45-0.92)** | **0.66 (0.46-0.95)** | 43 | 1,120 | 1.05 (0.59-1.85) | 0.94 (0.50-1.77) | 45 | 1,529 | **0.43 (0.27-0.69)** | **0.46 (0.28-0.76)** |
| ***Meat and eggs ≥ 5 times/week*** | | | | | | | | | | | | |
| No meat or amount of meat ≥ the size of 2 ping-pong balls and ≥ 1 egg | 113 | 2,935 | 1.00 | 1.00 | 51 | 1,383 | 1.00 | 1.00 | 62 | 1,552 | 1.00 | 1.00 |
| Amount of meat < the size of 2 ping-pong balls and < 1 egg | 16 | 387 | 1.08 (0.64-1.84) | 1.00 (0.59-1.71) | 7 | 140 | 1.34 (0.61-2.95) | 1.05 (0.46-2.42) | 9 | 246 | 0.96 (0.48-1.94) | 0.92 (0.45-1.89) |
| ***Seafood ≥ 3 times/week*** | | | | | | | | | | | | |
| No | 84 | 1,865 | 1.00 | 1.00 | 38 | 854 | 1.00 | 1.00 | 46 | 1,012 | 1.00 | 1.00 |
| Yes | 50 | 1,630 | 0.70 (0.50-1.00) | 0.71 (0.50-1.02) | 22 | 713 | 0.71 (0.42-1.21) | 0.74 (0.43-1.29) | 28 | 917 | 0.70 (0.44-1.11) | 0.74 (0.46-1.21) |
| ***Tofu or soy milk ≥ 3 times/week*** | | | | | | | | | | | | |
| No | 77 | 1,788 | 1.00 | 1.00 | 40 | 753 | 1.00 | 1.00 | 37 | 1,035 | 1.00 | 1.00 |
| Yes | 57 | 1,707 | 0.80 (0.57-1.13) | 0.77 (0.54-1.10) | 20 | 814 | **0.48 (0.28-0.83)** | **0.44 (0.25-0.78)** | 37 | 894 | 1.17 (0.74-1.84) | 1.24 (0.77-2.02) |
| ***Vegetables, seaweed, mushrooms (except kimchi) every meal*** | | | | | | | | | | | | |
| No | 60 | 1,852 | 1.00 | 1.00 | 28 | 801 | 1.00 | 1.00 | 32 | 1,051 | 1.00 | 1.00 |
| Yes | 74 | 1,643 | 1.36 (0.97-1.92) | 1.27 (0.89-1.82) | 32 | 766 | 1.18 (0.71-1.97) | 1.15 (0.66-2.00) | 42 | 878 | 1.53 (0.97-2.43) | 1.45 (0.90-2.33) |
| ***Fruits ≥ 5 days/week*** | | | | | | | | | | | | |
| No | 65 | 1,200 | 1.00 | 1.00 | 31 | 556 | 1.00 | 1.00 | 34 | 644 | 1.00 | 1.00 |
| Yes | 67 | 2,286 | **0.56 (0.40-0.79)** | **0.51 (0.35-0.73)** | 28 | 1,006 | **0.51 (0.30-0.85)** | **0.38 (0.21-0.69)** | 39 | 1,280 | **0.61 (0.38-0.96)** | 0.62 (0.37-1.02) |
| ***Milk or Dairy products ≥ 5 days/week*** | | | | | | | | | | | | |
| No | 97 | 2,260 | 1.00 | 1.00 | 42 | 1,131 | 1.00 | 1.00 | 55 | 1,129 | 1.00 | 1.00 |
| Yes | 35 | 1,226 | 0.70 (0.47-1.03) | **0.65 (0.44-0.97)** | 17 | 431 | 1.09 (0.62-1.92) | 1.09 (0.61-1.94) | 18 | 795 | **0.50 (0.29-0.85)** | **0.45 (0.26-0.79)** |
| ***≥ 3 side dishes (except soup or kimchi) at every meal*** | | | | | | | | | | | | |
| No | 26 | 918 | 1.00 | 1.00 | 11 | 379 | 1.00 | 1.00 | 15 | 540 | 1.00 | 1.00 |
| Yes | 106 | 2,567 | 1.44 (0.94-2.21) | 1.37 (0.88-2.15) | 48 | 1,183 | 1.39 (0.72-2.68) | 1.44 (0.71-2.94) | 58 | 1,384 | 1.47 (0.83-2.60) | 1.29 (0.70-2.37) |
| ***Taste salty when eating out*** | | | | | | | | | | | | |
| No | 54 | 1,540 | 1.00 | 1.00 | 24 | 630 | 1.00 | 1.00 | 30 | 910 | 1.00 | 1.00 |
| Yes | 76 | 1,924 | 1.14 (0.80-1.62) | 1.08 (0.76-1.54) | 35 | 931 | 0.98 (0.58-1.66) | 1.01 (0.58-1.76) | 41 | 993 | 1.28 (0.80-2.05) | 1.07 (0.65-1.76) |
| ***Tend to eat salty food*** | | | | | | | | | | | | |
| Yes | 12 | 383 | 1.00 | 1.00 | 3 | 157 | 1.00 | 1.00 | 9 | 226 | 1.00 | 1.00 |
| Neutral (Medium) | 96 | 2,341 | 1.33 (0.73-2.42) | 1.35 (0.73-2.50) | 44 | 1,091 | 2.00 (0.62-6.46) | 2.09 (0.61-7.20) | 52 | 1,249 | 1.10 (0.54-2.23) | 1.24 (0.59-2.57) |
| No | 24 | 761 | 1.04 (0.52-2.08) | 0.99 (0.49-2.01) | 12 | 313 | 1.93 (0.54-6.89) | 1.81 (0.48-6.86) | 12 | 448 | 0.73 (0.31-1.72) | 0.79 (0.33-1.91) |
| ***Grilled meat frequency*** | | | | | | | | | | | | |
| Never | 90 | 2,021 | 1.00 | 1.00 | 39 | 868 | 1.00 | 1.00 | 51 | 1,152 | 1.00 | 1.00 |
| Sometimes | 38 | 1,212 | 0.71 (0.48-1.04) | 0.72 (0.49-1.06) | 19 | 625 | 0.67 (0.39-1.18) | 0.66 (0.37-1.17) | 19 | 587 | 0.74 (0.44-1.25) | 0.81 (0.47-1.39) |
| Often | 3 | 233 | 0.28 (0.09-0.88) | 0.33 (0.10-1.04) | 1 | 68 | 0.29 (0.04-2.20) | 037 (0.04-3.09) | 2 | 165 | 0.27 (0.07-1.12) | 0.30 (0.07-1.25) |
| N/A (Not applicable) | 1 | 20 | 1.03 (0.14-7.46) | 1.67 (0.22-12.71) | 0 | 0 | NA | NA | 1 | 20 | 1.06 (0.15-7.68) | 1.82 (0.23-14.19) |

Model 1 was adjusted for age and sex. Age variable was excluded in age subgroups.

Model 2 was adjusted for age, sex, BMI, smoking status, household income, occupation. Age variable was excluded in age subgroups.

**Supplementary Table S6.** Association between TC risk and sex and smoking status

| **Exposure** | **TC cases** | **Person-years** | **HR (95% CI) for Model 1** | **HR (95% CI) for Model 2** |
| --- | --- | --- | --- | --- |
| ***Sex*** | | | | |
| Men | 18 | 36,695 | 1.00 | 1.00 |
| Women | 120 | 66,917 | **3.47 (2.11-5.71)** | **2.95 (1.49-5.84)** |
| ***Smoking status*** | | | | |
| Ever smoker | 23 | 33,862 | 1.00 | 1.00 |
| Never smoker | 114 | 69,520 | 1.04 (0.58-1.88) | 1.03 (0.57-1.87) |

Model 1 was adjusted for age. Sex was additionally adjusted in Model 1 of smoking status variable.

Model 2 was adjusted for age, BMI, household income, occupation. Smoking status was additionally adjusted in Model 2 of sex variable,

Sex was additionally adjusted in Model 2 of smoking status variable.
